# Dual anti-platelet therapy following percutaneous coronary intervention in a population of patients with thrombocytopenia at baseline: a meta-analysis

**DOI:** 10.1186/s40360-020-00409-2

**Published:** 2020-04-25

**Authors:** Manyun Long, Ziliang Ye, Jing Zheng, Wuxian Chen, Lang Li

**Affiliations:** Department of Cardiology, the First Affiliated Hospital of Guangxi Medical University, Guangxi Cardiovascular institute, Nanning, Guangxi 530021 P.R. China

**Keywords:** Percutaneous coronary intervention, Cardiovascular diseases, Thrombocytopenia, Low platelets, Bleeding, Stent thrombosis, Hemorrhagic stroke, Dual anti-platelet therapy

## Abstract

**Background:**

In this meta-analysis, we aimed to systematically compare the post percutaneous coronary interventional (PCI) adverse bleeding events, stent thrombosis, stroke and other cardiovascular outcomes in a population of patients with and without thrombocytopenia at baseline who were followed up on dual antiplatelet therapy (DAPT).

**Methods:**

Relevant English language articles which were published before June 2019 were retrieved from MEDLINE, http://www.ClinicalTrials.com, EMBASE, Cochrane central, and Google scholar briefly using specific terms such as percutaneous coronary intervention or dual antiplatelet therapy, and thrombocytopenia. All the participants were followed up on DAPT following discharge. Specific endpoints including bleeding events, stent thrombosis, stroke and other adverse cardiovascular events were assessed. The latest version of the RevMan software was used for the statistical assessment. Odd ratios (OR) with 95% confidence intervals (CI) based on a fixed or a random statistical model were used to represent the data graphically.

**Results:**

A total number of 118,945 participants (from 8 studies) were included with 37,753 suffering from thrombocytopenia at baseline. Our results showed post procedural bleeding (OR: 1.89, 95% CI: 1.16–3.07; *P* = 0.01), access site bleeding (OR: 1.66, 95% CI: 1.15–2.39; *P* = 0.006), intra-cranial bleeding (OR: 1.78, 95% CI: 1.30–2.43; *P* = 0.0003), gastro-intestinal bleeding (OR: 1.44, 95% CI: 1.14–1.82; *P* = 0.002) and any major bleeding (OR: 1.67, 95% CI: 1.42–1.97; *P* = 0.00001) to be significantly higher in thrombocytopenic patients treated with DAPT after PCI. Total stroke (OR: 1.45, 95% CI: 1.18–1.78; *P* = 0.0004) specifically hemorrhagic stroke (OR: 1.67, 95% CI: 1.30–2.14; *P* = 0.0001) was also significantly higher in these patients with thrombocytopenia at baseline. All-cause mortality and major adverse cardiac events were also significantly higher. However, overall total stent thrombosis (OR: 1.18, 95% CI: 0.90–1.55; *P* = 0.24) including definite and probable stent thrombosis were not significantly different compared to the control group.

**Conclusions:**

According to the results of this analysis, DAPT might have to be cautiously be used following PCI in a population of patients with thrombocytopenia at baseline due to the significantly higher bleeding rate including gastro-intestinal, intra-cranial bleeding and hemorrhagic stroke. Hence, special care might have to be taken when considering anti-platelet agents following PCI in these high risk patients. However, considering the present limitations of this analysis, this hypothesis will have to be confirmed in future trials.

## Background

Management of cardiovascular diseases (CVD) or acute coronary syndrome (ACS) in patients with thrombocytopenia has not often been an easy task for physicians due to the associated bleeding risks [1]. Anticoagulants and antiplatelet agents during and after percutaneous coronary intervention (PCI) respectively, are vital in patients with CVD [2, 3]. However, in this particular category of patients with a low platelet count at baseline, the use of dual antiplatelet therapy (DAPT) with aspirin and a P2Y12 inhibitor might be a risk [4]. The management of such patients with antiplatelet agents is challenging especially for physicians considering the fact that no current guidelines or recommendations are available to clearly and precisely guide physicians about how to manage patients with thrombocytopenia following PCI [5, 6].

This high risk group of patients has not yet well been investigated. Several reasons including a lack of evidence for the treatment and management of patients with thrombocytopenia following coronary angioplasty, as well as the statement in an editorial implying that nothing can be done to reduce bleeding risk in this category of patients following PCI [7], and the request for future randomized trials to evaluate the positive and negative effects of different antiplatelet agents or regimens on this category of patients, are the proofs for this ignorance by the research community till date.

In this meta-analysis, we aimed to systematically compare the post PCI adverse bleeding events, stent thrombosis, stroke and other cardiovascular outcomes in a population of patients with and without thrombocytopenia at baseline who were followed up on DAPT.

## Methods

### Sources of data

Articles which were published before June 2019 were retrieved from the following electronic search databases: MEDLINE, http://www.ClinicalTrials.com, EMBASE, Cochrane central, and Google scholar.

Furthermore, reference lists of publications which were relevant to coronary angioplasty in patients with thrombocytopenia at baseline were filtered for other suitable articles.

### Search strategies

Specific words or phrases were used to search for publications matching the scope of this current article:

Percutaneous coronary intervention AND thrombocytopenia OR low platelet counts; Dual anti-platelet therapy OR DAPT AND thrombocytopenia OR low platelet counts; Acute coronary syndrome OR ACS AND thrombocytopenia OR low platelet counts; Coronary angioplasty OR PCI AND thrombocytopenia OR low platelet counts.

Our search was restricted only to papers which were published in English language.

### Selection criteria (Inclusion and exclusion criteria)

We included studies based on the following criteria:

- They were studies (randomized trials or observational cohorts including prospective and retrospective studies) comparing PCI in patients with and without thrombocytopenia;
- They were not systematic reviews, meta-analyses, literature reviews, or letters of correspondence;
- Adverse bleeding events, stent thrombosis and/or adverse cardiovascular outcomes were reported among the endpoints;
- They involved patients who were treated by DAPT following PCI;
- They were published in English language;
- They involved patients with thrombocytopenia at baseline. It should be noted that studies involving thrombocytopenia which occurred after PCI were excluded from this analysis.

We excluded studies based on the following criteria:

- They were duplicated studies which were obtained from different search databases;
- They were literature reviews, meta-analyses, systematic reviews or case studies;
- They did not include participants with thrombocytopenia at baseline;
- They were published in another language apart from English;
- They did not report relevant adverse clinical outcomes.

### Definitions and endpoints to be assessed

In this analysis, thrombocytopenia was defined as a platelet count < 150,000 cells/μl.

This analysis is based on the assessment of bleeding events, stent thrombosis, stroke and other adverse cardiovascular outcomes in patients with thrombocytopenia at baseline who underwent PCI and who were followed up on DAPT post procedure.

The endpoints which were reported in the original studies have been listed in Table 1.

**Table 1:** Reported outcomes to be assessed

Studies	Outcomes which were reported after PCI in the original studies respectively	Follow-up time period after PCI	Types of CAD
Ayoub 2018 [8]	Post-procedural hemorrhage, RBC transfusion, platelet transfusion, vascular complications, acute ischemic CVA, acute hemorrhagic CVA, cardiac tamponade, mortality	In-hospital	General population with CAD + chronic thrombocytopenia undergoing PCI
Ito 2018 [9]	Myocardial infarction, GUSTO moderate bleeding, GUSTO severe bleeding, all-cause death, cardiac death, ischemic stroke, all stroke, hemorrhagic stroke, definite stent thrombosis, definite/probable stent thrombosis, any coronary revascularization, intracranial bleeding, gastrointestinal bleeding, bleeding related to surgery, bleeding related to catheterization procedure	3 years	General population with CAD + thrombocytopenia undergoing PCI
Kiviniemi 2013 [10]	All-cause mortality, MACCE, stroke, peripheral arterial embolism, myocardial infarction, revascularization, stent thrombosis, venous thromboembolism, total bleeding events, minor BARC 2 bleeding, major BARC bleeding (3a, 3b, 3c, 5)	1 year	CAD and atrial fibrillation + thrombocytopenia undergoing PCI
Liu 2018 [11]	All-cause mortality, cardiac death, myocardial infarction, revascularization, bleeding, major bleeding, ischemic stroke, MACE, definite stent thrombosis, definite and probable stent thrombosis, early, late and very late stent thrombosis	30 months	General population with CAD + thrombocytopenia undergoing elective PCI
Overgaard 2008 [12]	Mortality, MACE, myocardial infarction, major bleeding, gastro-intestinal bleed, intracranial hemorrhage, other bleedings, access site complications, transfusion	In-hospital	General population with CAD + thrombocytopenia undergoing PCI
Raphael 2016 [13]	Femoral bleeding, intra-cerebral bleeding, hematoma, gastro-intestinal bleeding, retroperitoneal bleeding, any bleeding, death, myocardial infarction, access site bleeding, BARC 2 minor bleeding, BARC (3a, 3b, 4) major bleeding	In-hospital	General population with CAD + thrombocytopenia undergoing PCI
Shiraishi 2019 [14]	Mortality, transfusion, access site bleeding	In-hospital	General population with CAD + thrombocytopenia undergoing elective PCI
Yadav 2016 [15]	Death, cardiac death, myocardial infarction, revascularization, definite/probable stent thrombosis, MACE	1 year	ACS (STEMI and NSTEMI) + thrombocytopenia undergoing PCI

*Abbreviations*: *PCI* percutaneous coronary intervention, *CAD* coronary artery disease, *ACS* acute coronary syndrome, *CVA* cerebrovascular attack, *RBC* red blood cells, *MACCE* major adverse cardiac and cerebrovascular events, *MACE* major adverse cardiac events, *BARC* bleeding defined according to the academic research consortium

The endpoints which were considered in this meta-analysis included:

(A) Endpoints assessing bleeding events:
  - Total bleeding events including any type of bleeding;
  - Post-procedural bleeding which was defined as bleeding complications immediately after the procedure;
  - Access site bleeding which was defined as bleeding occurring at the site which was accessible for intervention (radial or femoral);
  - Any minor bleeding (consisting of any type of minor bleeding);
  - Any major bleeding (consisting of any type of major bleeding);
  - Bleeding defined according to the academic research consortium (BARC) [16];

BARC type 1: was defined as minimal bleeding that does not require hospital assistance or treatment.

BARC type 2: was defined as any overt bleeding, that does not fit the criteria for type 3, 4 and 5, but does meet one of the following criteria: requiring medical or non-surgical intervention by a medical healthcare professional, or leading to hospitalization or increased level of care.

BARC type 3: was defined as overt bleeding with a hemoglobin drop of 3–5 g/dl, and resulting in blood transfusion.

BARC type 4: was defined as coronary artery bypass grafting related bleeding.

BARC type 5: was defined as fatal bleeding.

BARC type 1 and 2 were classified as BARC minor bleeding.

BARC type 3–5 were classified as BARC major bleeding.

(g) Intra-cranial bleeding;
(h) Gastro-intestinal bleeding.

(B) Endpoints assessing stent thrombosis:
  - Overall stent thrombosis;
  - Definite stent thrombosis;
  - Definite/probable stent thrombosis defined by the academic research consortium [17].
(C) Endpoints assessing stroke:
  - Total stroke including ischemic and hemorrhagic stroke;
  - Ischemic stroke;
  - Hemorrhagic stroke.

(D) Endpoints assessing adverse cardiovascular outcomes:
  - All-cause mortality;
  - Cardiac death;
  - Revascularization (including target vessel revascularization and/or target lesion revascularization);
  - Myocardial infarction (MI);
  - Major adverse cardiac events (MACEs): defined as the composite endpoint including mortality, MI, and revascularization. Since, one study reported major adverse cerebrovascular and cardiovascular events (MACCEs) consisting of all the components of MACEs with the addition of stroke, we have included this outcome along with MACEs.

The original studies had a follow-up time period ranging from an in-hospital follow-up to a time period of 3 years. The respective follow-up time periods have also been listed in Table 1.

### Data extraction and quality assessment

Tables in this paper consisted of vital data which were extracted from the original studies by the authors. Those data included the outcomes (bleeding events, stent thrombosis, stroke and other cardiovascular outcomes) reported, the follow-up time periods (in hospital and longer duration time period), DAPT which were used (drugs involved), the platelet count at baseline, the baseline characteristics of the participants including comorbidities and cardiac risk factors, the total number of participants with versus without thrombocytopenia and so on.

Disagreement which occurred during the data extraction process was resolved among the authors by consensus or by close discussion with the corresponding author. An online platform was set up for the authors to discuss any issue related to the data extraction process. Any disagreement was further discussed by the corresponding author, who was the last one to reach a final decision.

The Newcastle Ottawa Scale (NOS) [18] was used to assess the methodological quality of the observational cohorts whereas the criteria recommended by the Cochrane Collaboration [19] were used to assess the methodological quality of the randomized controlled trials. Grades from A to C were allotted denoting low, moderate and high risks of bias respectively.

### Statistical analysis

This analysis was carried out with the latest version of the RevMan software (RevMan 5.3). Odds ratios (OR) with 95% confidence intervals (CI) were used to represent the data graphically.

Heterogeneity assessment was carried out with the Q statistic test and a subgroup analysis with (*P* ≤ 0.05) was considered significant statistically. The I^2^ statistic test was also used to assess heterogeneity whereby an increase in its value represented an increasing heterogeneity.

Concerning the statistical model which was used: a fixed effect model was used if the heterogeneity value I^2^ was less than 50%. Or else, a random effect model was used.

In addition to this analysis, sensitivity analysis was also carried out based on an exclusion method.

Publication bias was visually observed by a careful assessment of the funnel plots.

### Ethical approval

No experiment involving animals or humans were carried out by any of the authors. Therefore, an ethical or board review approval was not required for this meta-analysis.

## Results

### Outcome of the search process and the selection of studies

Taking into consideration the PRISMA guideline [20], 742 publications were searched through electronic databases. The titles and abstracts were carefully assessed by the authors. Articles which were irrelevant were directly eliminated (654).

Eighty eight (88) full text articles were then assessed based on the eligibility criteria for selection.

Another set of elimination was carried out based on the following reasons:

- They were literature reviews (3);
- They were case studies (17);
- They were based on thrombocytopenia after PCI and did not involve participants with thrombocytopenia at baseline (26);
- They were letters of correspondence (4);
- They were duplicated studies from several different search databases (30).

**Fig. 1:**
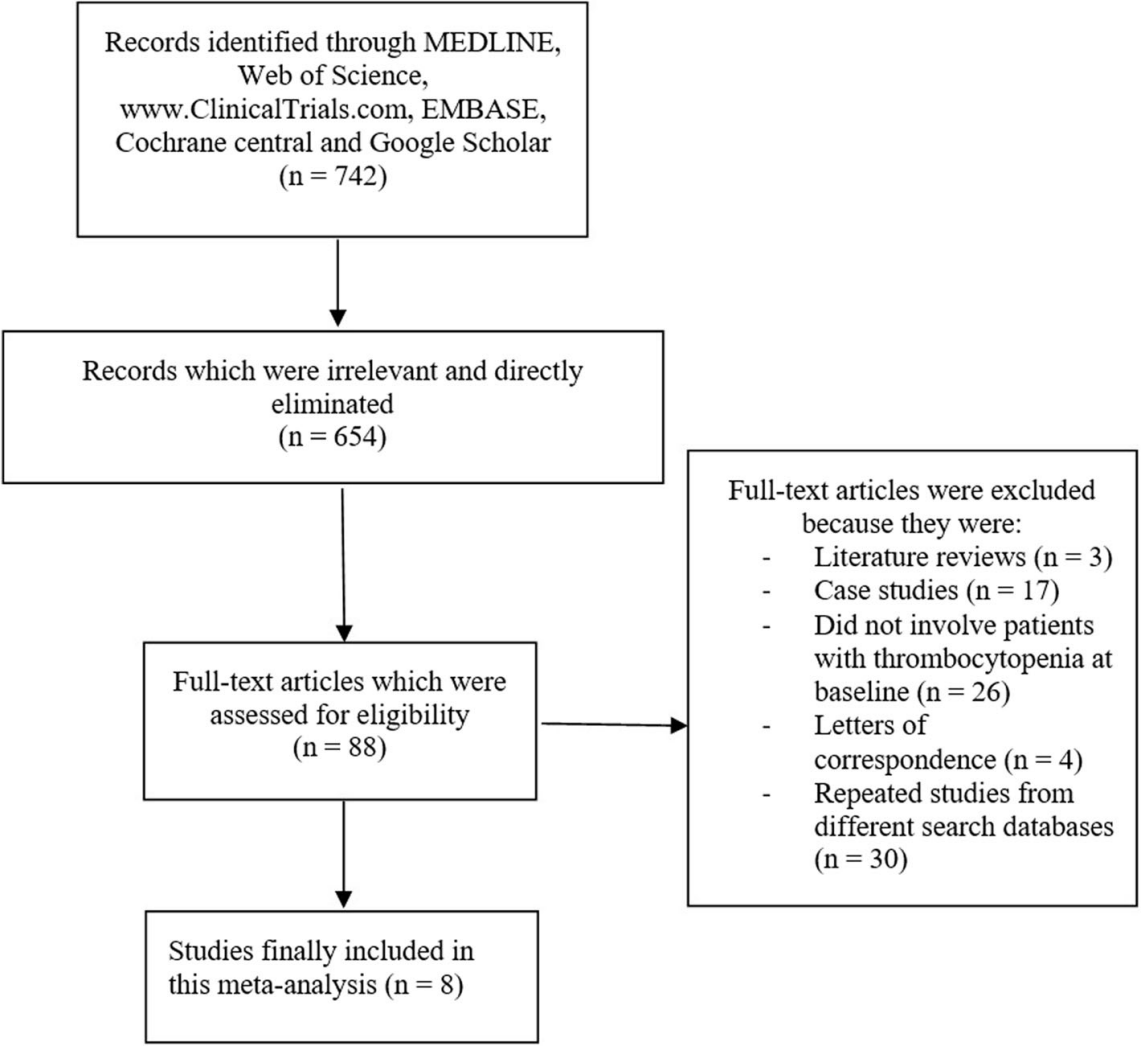
Flow diagram showing the selection of studies to be included in this meta-analysis

Finally, we were remaining with 8 studies [8–15] that satisfied all the criteria for inclusion and exclusion represented by Fig. 1.

### Main features of the studies

Seven studies were observational cohorts whereas one study involved data from a randomized trial. A total number of 118,945 participants were included in this meta-analysis. Thirty seven thousand seven hundred and fifty three (37,753) participants suffered from thrombocytopenia at baseline whereas 81,192 participants were non-thrombocytopenic participants who underwent PCI. The detailed list has been given in Table 2.

**Table 2:** Main features of the studies

Studies	Type of study	Methodological quality grading	No of patients with thrombocytopenia at baseline (n)	No of patients in the control group (n)	Average PLT count in the study group
Ayoub 2018 [8]	OS	B	32, 565	32, 565	< 150,000 cells/μl
Ito 2018 [9]	OS	B	2298	16, 763	100–150,000 cells/μl
Kiviniemi 2013 [10]	OS	B	99	762	80–150,000 cells/μl
Liu 2018 [11]	OS	B	1263	8634	50–150,000 cells/μl
Overgaard 2008 [12]	OS	B	639	10,182	< 150,000 cells/μl
Raphael 2016 [13]	OS	B	146	1281	≤ 100,000 cells/μl
Shiraishi 2019 [14]	OS	B	226	1009	50,000–149,000 cells/μL
Yadav 2016 [15]	RCT	B	607	9996	< 150,000 cells/μl
Total number of participants (n)			37,753	81,192	

*Abbreviations*: *PLT* platelet, *OS* observational study, *RCT* randomized controlled trials

The definition of thrombocytopenia reported in each study has also been given in Table 2.

After an assessment of the studies, a grade B was allotted (moderate risk of bias) to each of the original study.

### Baseline features of the participants

The baseline characteristics of the participants with and without thrombocytopenia have been listed in Table 3. The mean age (57.9–74.0 years), the mean percentage of participants who were males, who suffered from diabetes mellitus, hypertension, dyslipidemia and who had a history of smoking have been listed in Table 3.

**Table 3:** Baseline characteristics

Studies	Age	Males	T2DM	HTN	Smoking	DYS
	TC/NTC	TC/NTC	TC/NTC	TC/NTC	TC/NTC	TC/NTC
Ayoub 2018 [8]	68.8/69.3	73.1/73.9	45.9/46.9	77.1/77.9	–	–
Ito 2018 [9]	71.8/68.0	79.5/72.9	44.4/39.6	81.5/81.8	21.2/28.6	39.7/42.3
Kiviniemi 2013 [10]	74.0/73.0	85.0/68.0	31.0/37.0	76.0/85.0	6.00/11.0	69.0/66.0
Liu 2018 [11]	60.9/57.9	13.5/24.5	35.2/29.3	60.9/64.8	59.3/56.2	65.5/67.5
Overgaard 2008 [12]	67.0/63.0	84.5/71.5	32.2/26.9	61.7/61.3	46.0/38.0	–
Raphael 2016 [13]	70.6/67.4	82.0/71.0	44.0/30.0	85.0/80.0	–	84.0/85.0
Shiraishi 2019 [14]	74.0/72.0	74.8/73.4	53./43.8	77.4/75.9	11.9/16.2	60.6/66.5
Yadav 2016 [15]	65.7/61.9	87.3/73.8	32.0/23.5	67.7/61.1	26.9/36.0	57.9/51.5

*Abbreviations*: *T2DM* type 2 diabetes mellitus, *HTN* hypertension, *DYS* dyslipidemia, *TC* thrombocytopenia group, *NTC* non-thrombocytopenia group

In addition, the anticoagulants or antiplatelet drugs which were used during this invasive procedure and which were prescribed at discharge following PCI were listed in Table 4.

**Table 4:** Intra-procedural and post angioplasty anticoagulants and anti-platelets used

Studies	Intra-procedural anti-platelets/anti-coagulants	Post procedural/discharge anti-platelets (majority)	Duration of DAPT use	Access site for PCI
Ayoub 2018 [8]	Not mentioned	Not mentioned	Not mentioned	Not mentioned
Ito 2018 [9]	Unfractionated heparin	Aspirin, clopidogrel or ticlopidine (DAPT)	≥ 3 months	Not mentioned
Kiviniemi 2013 [10]	LMWH (enoxaparin sodium and dalteparin), unfractionated heparin, bivalirudin, and glycoprotein IIb/IIIa inhibitors	Aspirin, clopidogrel and warfarin Or Aspirin and clopidogrel (DAPT)	1–3 months	Femoral access
Liu 2018 [11]	Aspirin, clopidogrel, LMWH, glycoprotein IIb/IIIa inhibitors	Aspirin and clopidogrel (DAPT)	short term use without mentioning the exact time duration	Not mentioned
Overgaard 2008 [12]	Not mentioned	Not mentioned	Not mentioned	Femoral access
Raphael 2016 [13]	Aspirin, clopidogrel, heparin	Aspirin and clopidogrel (DAPT)	Not mentioned	Femoral > radial access
Shiraishi 2019 [14]	Aspirin, thienopyridine	Aspirin, clopidogrel, ticlopidine or prasugrel (DAPT)	Not mentioned	Radial > femoral access
Yadav 2016 [15]	Heparin, bivalirudin, and glycoprotein IIb/IIIa inhibitors, aspirin and clopidogrel	Aspirin, clopidogrel, ticlopidine or prasugrel (DAPT)	Not mentioned	Not mentioned

*Abbreviations*: *LMWH* low molecular weight heparin, *DAPT* dual antiplatelet therapy, *PCI* percutaneous coronary intervention

### Main clinical endpoints which were assessed

**Fig. 2:**
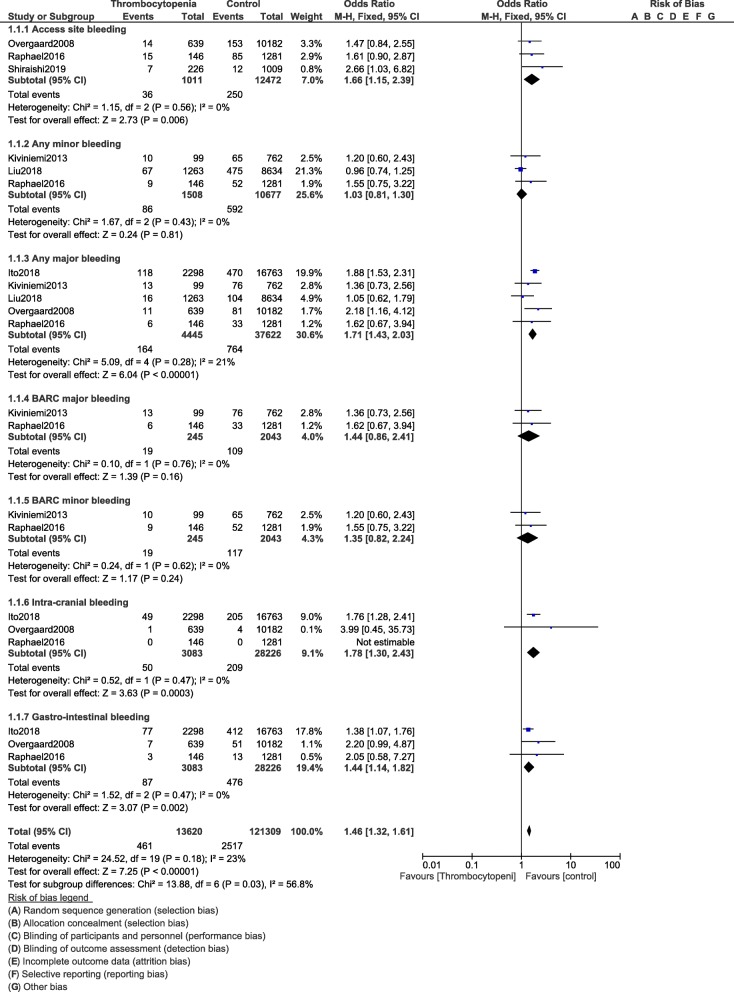
Bleeding events following PCI in these patients with thrombocytopenia at baseline and on dual antiplatelet therapy (Part I)

Our results showed access site bleeding (OR: 1.66, 95% CI: 1.15–2.39; *P* = 0.006), any major bleeding (OR: 1.67, 95% CI: 1.42–1.97; *P* = 0.00001), intra-cranial bleeding (OR: 1.78, 95% CI: 1.30–2.43; *P* = 0.0003) and gastro-intestinal bleeding (OR: 1.44, 95% CI: 1.14–1.82; *P* = 0.002) to be significantly higher in this category of patients following PCI as shown in Fig. 2. Any minor bleeding (OR: 1.03, 95% CI: 0.81–1.30; *P* = 0.81), BARC minor bleeding (OR: 1.35, 95% CI: 0.82–2.24; *P* = 0.24) and BARC major bleeding (OR: 1.44, 95% CI: 0.86–2.41; *P* = 0.16) were not significantly different as shown in Fig. 2.

**Fig. 3:**
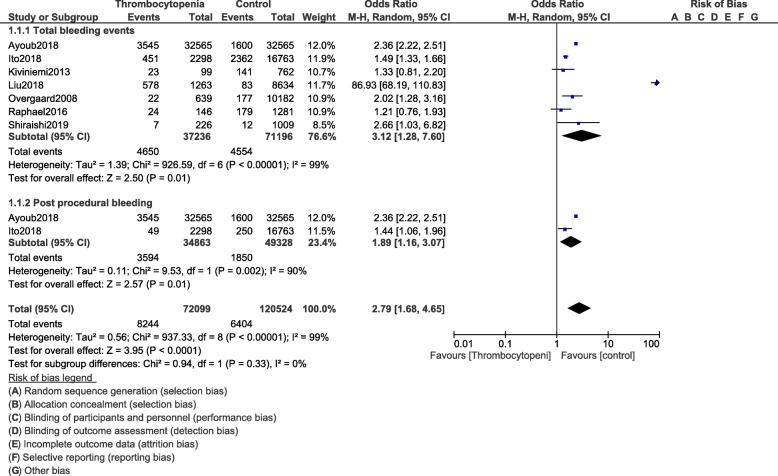
Bleeding events following PCI in these patients with thrombocytopenia at baseline and on dual antiplatelet therapy (Part II)

**Fig. 4:**
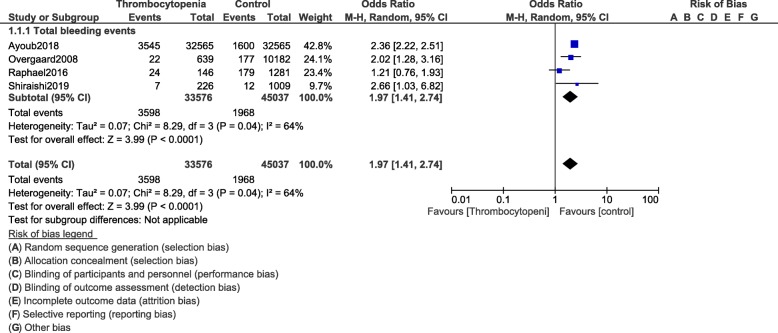
In Hospital bleeding events following PCI in these patients with thrombocytopenia at baseline and on dual antiplatelet therapy

However, when total bleeding event was assessed, it was significantly higher (OR: 3.12, 95% CI: 1.28–7.60; *P* = 0.01) in the population of patients with thrombocytopenia at baseline as shown in Fig. 3. Total in hospital bleeding (OR: 1.97, 95% CI: 1.41–2.74; *P* = 0.0001) was also significantly higher (Fig. 4). Post-procedural bleeding (OR: 1.89, 95% CI: 1.16–3.07; *P* = 0.01) was also significantly higher in these patients with thrombocytopenia following coronary stenting as shown in Fig. 3.

**Fig. 5:**
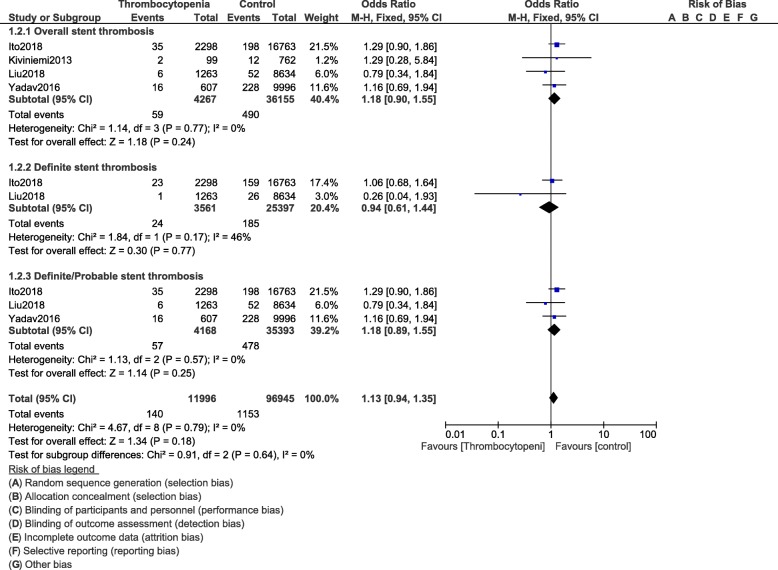
Stent thrombosis following PCI in these patients with thrombocytopenia at baseline and on dual antiplatelet therapy

Our results showed overall total stent thrombosis (OR: 1.18, 95% CI: 0.90–1.55; *P* = 0.24), definite stent thrombosis (OR: 0.94, 95% CI: 0.61–1.44; *P* = 0.77) and definite/probable stent thrombosis (OR: 1.18, 95% CI: 0.89–1.55; *P* = 0.25) not to be significantly different in patients with thrombocytopenia compared to the control group as shown in Fig. 5.

**Fig. 6:**
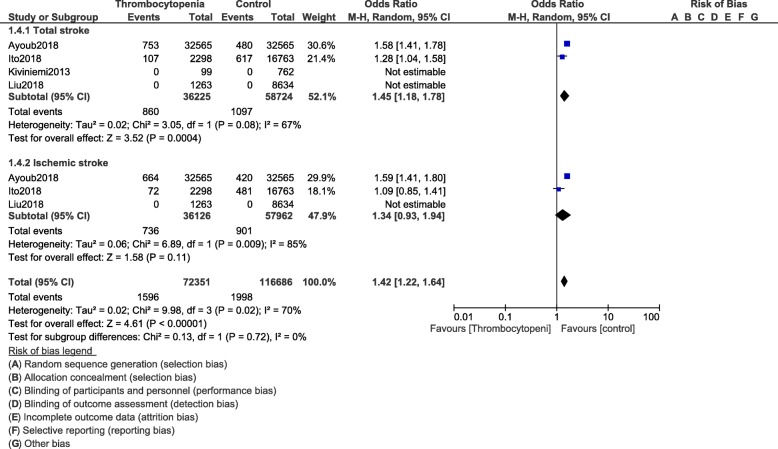
Stroke following PCI in these patients with thrombocytopenia at baseline and on dual antiplatelet therapy (Part I)

**Fig. 7:**
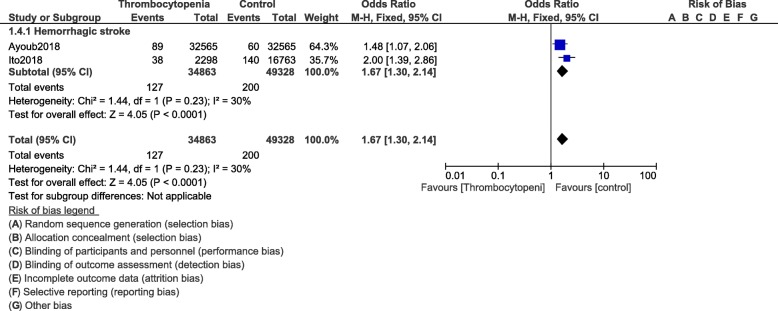
Stroke following PCI in these patients with thrombocytopenia at baseline and on dual antiplatelet therapy (Part II)

When stroke was assessed, following PCI, total stroke (OR: 1.45, 95% CI: 1.18–1.78; *P* = 0.0004) was significantly higher in these patients with thrombocytopenia as shown in Fig. 6. Our results showed hemorrhagic stroke (OR: 1.67, 95% CI: 1.30–2.14; *P* = 0.0001) to be significantly higher in these patients with thrombocytopenia as shown in Fig. 7. Ischemic stroke (OR: 1.34, 95% CI: 0.93–1.94; *P* = 0.11) was similarly manifested (Fig. 6).

**Fig. 8:**
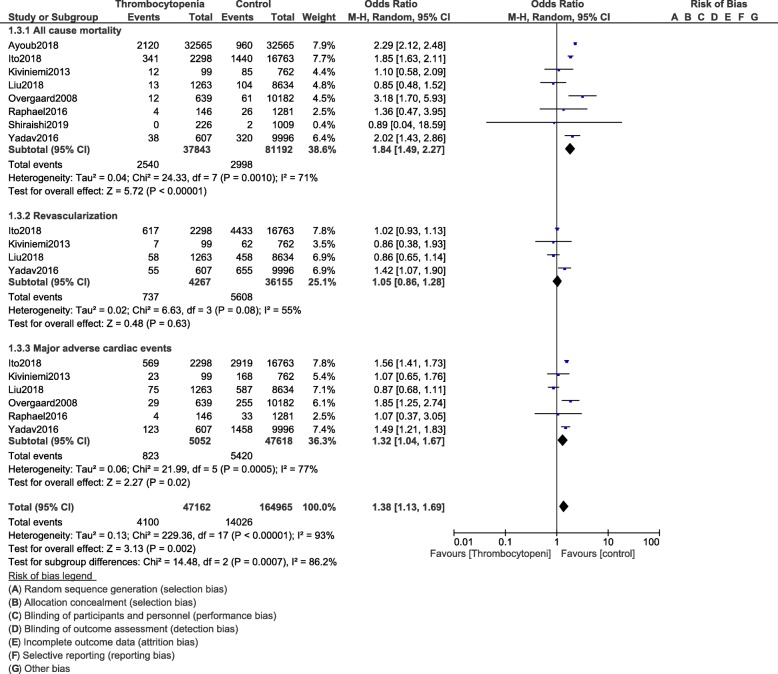
Cardiovascular outcomes following PCI in these patients with thrombocytopenia at baseline and on dual antiplatelet therapy (Part I)

**Fig. 9:**
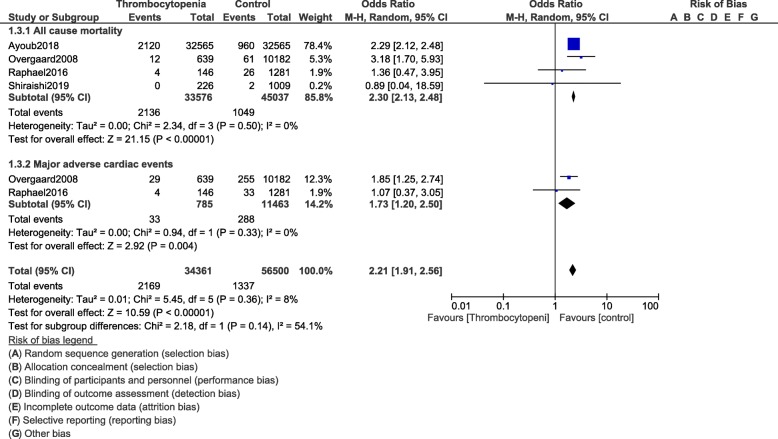
In hospital cardiovascular outcomes following PCI in these patients with thrombocytopenia at baseline and on dual antiplatelet therapy

**Fig. 10:**
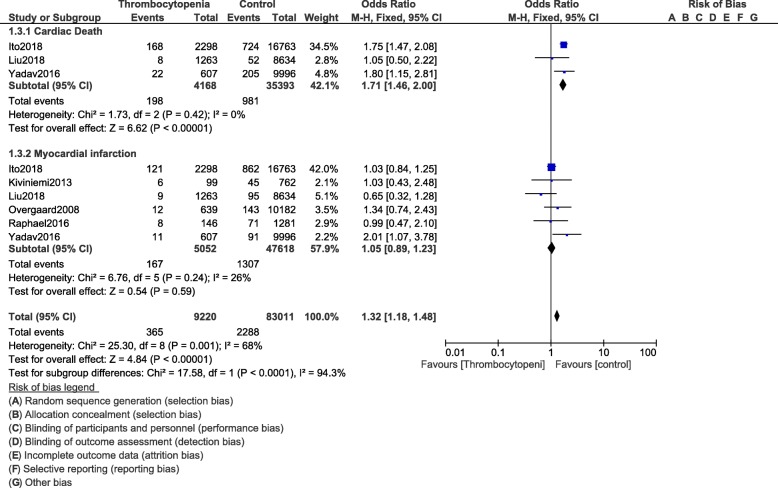
Cardiovascular outcomes following PCI in these patients with thrombocytopenia at baseline and on dual antiplatelet therapy (Part II)

All-cause mortality (OR: 1.84, 95% CI: 1.49–2.27; *P* = 0.00001) and MACEs (OR: 1.32, 95% CI: 1.04–1.67; *P* = 0.02) were significantly higher in the population of patients with thrombocytopenia as shown in Fig. 8. In hospital mortality (OR: 2.30, 95% CI: 2.13–2.48; *P* = 0.00001) and in hospital MACEs (OR: 1.73, 95% CI: 1.20–2.50; *P* = 0.004) were also significantly higher (Fig. 9). Cardiac death (OR: 1.71, 95% CI: 1.46–2.00; *P* = 0.00001) was also significantly higher. However, revascularization (OR: 1.05, 95% CI: 0.86–1.28; *P* = 0.63) and MI (OR: 1.05, 95% CI: 0.89–1.23; *P* = 0.59) were similar in both the experimental and the control groups as shown in Figs. 8 and 10.

**Fig. 11:**
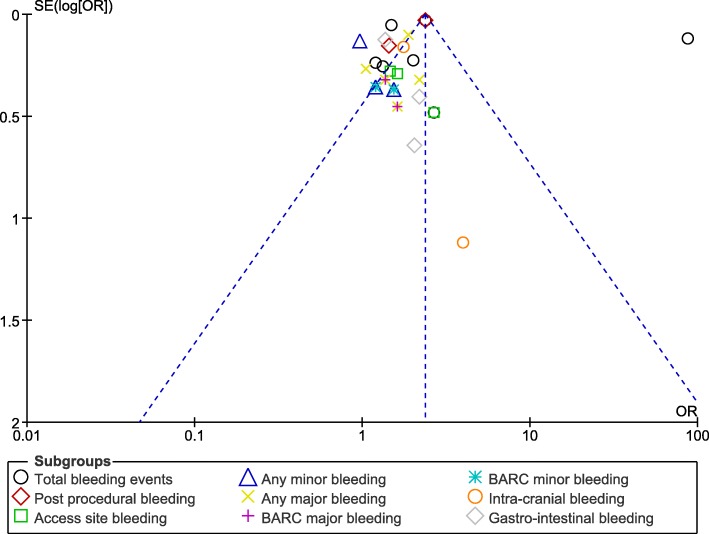
Funnel plot representing any evidence of publication bias (A)

**Fig. 12:**
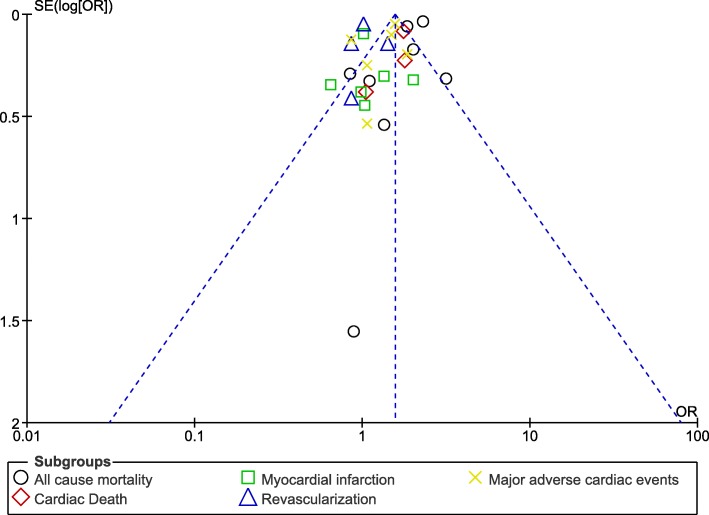
Funnel plot representing any evidence of publication bias (B)

Moreover, in order for the final results not to be influenced by any particular study, a sensitivity analysis was carried out. During this sensitivity analysis, consistent results were obtained throughout. Also, the visual assessment of publication bias was shown in Figs. 11 and 12.

## Discussion

Thrombocytopenia is a rare disorder that affects a minority of patients all around the globe. Due to a low platelet count, these patients are at higher risk for bleeding complications [21]. The use of anticoagulants during and antiplatelet agents after PCI in thrombocytopenic patients with ACS is still controversial and is a dilemma among clinical physicians. Even though platelets have a major contribution in the pathogenesis and occurrence of ACS [22], low platelet counts in thrombocytopenia does not reduce this problem. In contrast, platelets are often larger and hyperactive despite being low in number in these patients with thrombocytopenia.

As described in the result section, several types of bleeding including post-procedural bleeding, access site bleeding, intra-cranial bleeding, gastro-intestinal bleeding and any major bleeding were significantly higher in this population of patients with thrombocytopenia following the use of DAPT after PCI. The overall bleeding risk was high. Hemorrhagic stroke was also higher in this category of patients. However, our analysis showed stent thrombosis to be similar in patients with and without thrombocytopenia who were followed up on DAPT after PCI.

When the other cardiovascular outcomes including all-cause mortality, cardiac death and MACEs were assessed, a significantly higher risk was observed following PCI in these patients with thrombocytopenia at baseline. It should be noted that this high mortality rate might have partly resulted from the significantly higher risks of overall and major bleeding as well as hemorrhagic stroke. In addition, significant bleeding might have resulted in severe MI resulting in a significantly higher level of cardiac death in this category of patients.

The effect of DAPT was further demonstrated in a case study [23] of a 77 year old patient with a history of thrombocytopenia at baseline undergoing PCI with the implantation of drug eluting stents. He was followed up on DAPT with aspirin and ticagrelor. However, during the third day of hospitalization, several episodes of epistaxis were noted, due to which, DAPT was stopped, and the patient was discharged on clopidogrel mono-therapy. After 1 year, the patient was reported to be well, without any bleeding event, even with a platelet count of 31–60 X 10 ^9^/L.

In a letter of correspondence [24] based on the medical and interventional management of patients with severe thrombocytopenia (platelets < 50 X 10 ^9^/L) undergoing PCI, the authors stated that PCI is not recommended in patients with severe thrombocytopenia due to the high risk of bleeding events because of the use of peri-procedural anticoagulants and post-procedural DAPT, hence, data were collected from 35 participants from January 2006 to December 2010 with severe thrombocytopenia at baseline undergoing coronary angioplasty to show the clinical complications and management in these patients. Radial access was considered in these patients to minimize the risk of bleeding events. Among the 35 participants, drug eluting stents were used in only 5 patients while in the remaining, bare metal stents were implanted. Unfractionated heparin was used in most of the patients during the procedure. After the procedure, clopidogrel mono-therapy was initiated in 20% of the 35 participants. However, after 7 months, the antiplatelet mono-therapy was discontinued in one patient due to increased bleeding complications. DAPT for 6 months was recommended in another 20% of the 35 participants, whereby only 5 participants completed the full course. MI was reported in 1 patient who was implanted with bare metal stents and taking aspirin mono-therapy due to stent thrombosis after 3 months of treatment. The authors stated that approximately 50% of the 35 patients with severe thrombocytopenia experienced bleeding complications. However, bleeding events were superficial, and involved gastro-intestinal and genitourinary bleeds. Among the 7 patients who were on DAPT, 3 patients suffered serious bleeding events, and among the 7 patients who were on clopidogrel mono-therapy, 1 patient experienced severe bleeding. The remaining participants were only on aspirin mono-therapy and only 2 participants reported severe bleeding complications. It should be noted that antiplatelet regimens were discontinued at the time of bleeding. Nevertheless, in this current analysis, where DAPT was used by the majority of the participants, higher bleeding events were observed and it should be noted that this current analysis also involved far more number of participants (over 10, 000 times more that the above mentioned study). While in the previous study, hemorrhagic bleed was not observed, our current analysis showed significantly higher risk of hemorrhagic stroke among those participants on DAPT after PCI.

Thrombocytopenia co-existing in patients with diseases such as liver cirrhosis, leukemia or patients who are on chemotherapy might be associated with higher risks for severe bleeding events following the use of DAPT. For example, following PCI in a patient with acute myeloid leukemia receiving chemotherapy [25], DAPT was prescribed but the duration was adapted based on the patient’s tolerability and circumstance. A personalized treatment strategy should be thought to maintain a balance between the effect and risk for bleeding [26]. Similarly, in cirrhotic patients with coronary artery disease, DAPT while reducing the risk of recurrent myocardial infarction, it was associated with a higher gastrointestinal bleeding [27] which might later result in discontinuation or modification of this antiplatelet regimen.

Optimal DAPT use in patients with ACS has always been a controversial issue. The PRECISE-DAPT and the PARIS risk scores were recently developed to facilitate the choice for individualized optimal DAPT use with aspirin and ticagrelor or prasugrel following PCI [28]. Four thousand four hundred twenty-four participants were followed up for 14 months upon discharge after revascularization by PCI. Major bleeding risk was stratified and data supported the use of the PRECISE-DAPT for such patients whereas the PARIS risk score was more appropriate for prediction in patients with respect to ischemic events.

Furthermore, in an individual data pairwise and network meta-analysis of six randomized trials and 11, 473 patients [29], the authors demonstrated that 3 months DAPT use appeared safe in stable coronary artery patients. The authors also concluded that prolonged DAPT use might increase bleeding risk regardless of clinical presentation. Our current analysis already involved participants with thrombocytopenia, and therefore, logically, longer duration of DAPT use might not be tolerable in similar patients following PCI. Another study showed short term DAPT use to be equally effective and safer compared to long term DAPT use following stents implantation with drug eluting stents [30].

This analysis was based on patients with thrombocytopenia at baseline, and who underwent PCI and who were followed up on DAPT. It should be noted that in this study, thrombocytopenia was already confirmed prior to PCI and not following the use of certain types of anticoagulants [31, 32].

### Limitations

This present meta-analysis based on PCI in patients with thrombocytopenia at baseline has certain limitations. First of all, most of the data were obtained from observational studies which might result in the introduction of bias and other confounding factors that might have affected the results. Secondly, the anticoagulants and antiplatelet agents which were used during the invasive procedure were not clearly stated. This is quite an important information which was not fully reported in the original papers and might be another vital limitation of this analysis. In addition, in two studies, the anti-platelet regimens after the procedure were not mentioned. Hence, we are not sure if DAPT was used or these patients were treated on mono-therapy following PCI. This could be another limitation of this study. Moreover, in one study, the participants were treated with aspirin and warfarin as the anticoagulants after PCI whereas all the other studies reported aspirin and P2Y12 inhibitors (mainly clopidogrel). Also, the definition of thrombocytopenia varied in some studies and this analysis included a mixture of mild and moderate thrombocytopenic patients. However, the range of platelet count which was stated in most of the studies was almost similar. Furthermore, access site bleeding which was one of the outcomes, was strictly dependent on the procedural access sites which were involved (radial or femoral). Normally a radial access is associated with lower bleeding risk in comparison to the femoral access. However, several original studies did not mention about which access sites were involved and this might be another limitation of this analysis. In addition, it should be noted that the causes of thrombocytopenia are major determinants of prognosis following PCI in these patients. However, in this study, we were unable to analyze prognosis on the basis of mechanism causing platelet deficit and for this reason the results on mortality might not be generalized and would require more dedicated studies. At last, the duration of DAPT use was also not reported in most of the original studies and this might also contribute to a major limitation of this study.

## Conclusions

According to the results of this analysis, DAPT might have to be cautiously be used following PCI in a population of patients with thrombocytopenia at baseline due to the significantly higher bleeding rate including gastro-intestinal, intra-cranial bleeding and hemorrhagic stroke. Hence, special care might have to be taken when considering anti-platelet agents following PCI in these high risk patients. However, considering the present limitations of this analysis, this hypothesis will have to be confirmed in future trials.

## Data Availability

All data and materials used in this research are freely available in electronic databases (MEDLINE, EMBASE, Cochrane database, Google scholar). References have been provided.

## Abbreviations

PCI: Percutaneous coronary intervention
MACEs: Major adverse cardiac events
ST: Stent thrombosis
OR: Odd ratios
DAPT: Dual anti-platelet therapy
